# Cannabidiol and Sertraline Regulate Behavioral and Brain Gene Expression Alterations in an Animal Model of PTSD

**DOI:** 10.3389/fphar.2021.694510

**Published:** 2021-06-28

**Authors:** Ani Gasparyan, Francisco Navarrete, Jorge Manzanares

**Affiliations:** ^1^ Instituto de Neurociencias, Universidad Miguel Hernández-CSIC, Alicante, Spain; ^2^ Red Temática de Investigación Cooperativa en Salud (RETICS), Red de Trastornos Adictivos, Instituto de Salud Carlos III, MICINN and FEDER, Madrid, Spain

**Keywords:** PTSD, mice model, cannabidiol, sertraline, mRNA

## Abstract

This study evaluated the effects of cannabidiol (CBD) and/or sertraline (STR) on behavioral and gene expression alterations induced by a new chronic animal model of post-traumatic stress disorder (PTSD). C57BL/6J male mice were repeatedly exposed to physical and psychogenic alternate stressful stimuli. Fear-related memory and anxiety-like behaviors were evaluated. The effects of the administration of CBD (20 mg/kg, i.p.) and/or STR (10 mg/kg, p.o.) were analyzed on behavioral and gene expression changes induced by the model of PTSD. Gene expression alterations of targets related with stress regulation, endocannabinoid and serotonergic systems were analyzed by real-time PCR. The results revealed an increased and long-lasting fear-related memory and anxiety-like behaviors in mice exposed to the animal model of PTSD. Treatment with CBD improved these behaviors in PTSD animals, effects that were significantly potentiated when combined with STR. Gene expression analyses revealed a long-term increase of corticotropin releasing factor (*Crf*) that was significantly normalized with the combination CBD plus STR. Cannabinoid receptors (*Cnr1* and *Cnr2*) were up regulated in PTSD mice whereas the serotonin transporter (*Slc6a4*) was reduced. Interestingly, CBD and STR alone or combined induced a significant and marked increase of *Slc6a4* gene expression. These results point out the cooperative action of the combination CBD plus STR to enhance fear extinction and reduce anxiety-like behaviors, normalizing gene expression alterations in this animal model of PTSD and suggesting that the combination of CBD with STR deserves to be further explored for the treatment of patients with PTSD.

## Introduction

Post-traumatic stress disorder (PTSD) is a disabling mental condition caused by the exposure to frightening or threatening life events (APA, 2013). Around a 70% of worldwide population experience one or more traumatic events in any moment of their lives, whereas 10–15% develop PTSD. Type, severity and number of traumatic events, associated with individual susceptibility or the stage of life in which the trauma occurs influences the likelihood of developing PTSD (Kessler et al., 2017).

It remains essential to identify new therapeutic targets that may improve PTSD treatment. From a translational point of view, it is crucial to identify animal models to recapitulate PTSD-related clinical traits by the exposure to different kind of stressors, mainly psychogenic (e.g., predator threat), physic (e.g., electric shock), and psychosocial (e.g., disturbances in housing conditions). However, it is unlikely that a single animal model will reproduce the complexity of the human disorder only mimicking core aspects of human PTSD such as fear dysregulation and increased anxiety-like behavior.

There is a broad range of multi-disciplinary experimental approaches to induce a PTSD-like syndrome (Daskalakis et al., 2013; Singewald and Holmes, 2019; Zhang et al., 2019). However, there is a need of chronic animal models of PTSD to induce intense and long-lasting (several weeks) emotional disturbances. These prolonged alterations will simulate more closely the time course of PTSD-related behavioral and neurochemical changes and, therefore, would permit to study the effects of chronic pharmacological treatments (3–5 weeks).

Currently approved medications for the treatment of PTSD are the selective serotonin reuptake inhibitors (SSRIs) paroxetine and sertraline (STR). These drugs present important limitations regarding the response rate that rarely exceeds 60%, and only 30% corresponds to complete remission (Berger et al., 2009). In addition, the available treatments present relevant side effects that may limit tolerance or even decrease therapeutic adherence (Shin et al., 2014). Therefore, there is an increasing need to develop new pharmacological strategies to improve the complex management of PTSD symptomatology. Interestingly, recent research advances revealed the pivotal role of the endocannabinoid system in the regulation of fear memory and emotional behavior in PTSD (Berardi et al., 2016). In this sense, cannabidiol (CBD) has attracted growing attention due to its lack of abuse potential (Viudez-Martinez et al., 2019), its multimodal mechanism of action (Elsaid and Le Foll, 2019) and especially its effects on the regulation of fear-related memories (Song et al., 2016). Indeed, several animal studies showed that CBD facilitates extinction, decreases retrieval or acquisition, and blocks reconsolidation of contextual fear memory evaluated in a fear conditioning (FC) paradigm (Bitencourt and Takahashi, 2018). Furthermore, human studies also suggested the therapeutic potential of CBD for the treatment of PTSD symptoms related with fear extinction, anxiety and sleep disturbances (Das et al., 2013; Shannon and Opila-Lehman, 2016; Elms et al., 2019). However, no previous studies have evaluated the effects of chronic CBD administration, alone or in combination with STR, on the behavioral and neurochemical impairments produced by an animal model of PTSD.

Therefore, the main goals of this study were: 1) to characterize and validate a long-lasting animal model of PTSD by exposing C57BL/6J adolescent mice to alternating and unpredictable psychogenic (fox urine), physic (electric shock, movement restriction) and psychosocial stressors (wet bedding, tilted cage, food deprivation) during a 5-weeks period, including two intermediate resting weeks to add a pivotal re-exposure factor for modeling PTSD, and 2) to evaluate the effects of repeated administration of CBD, STR, and CBD plus STR combination on behavioral and neurochemical alterations induced by this animal model of PTSD. Fear-related memory and anxiety-like behaviors were evaluated by the FC paradigm, and by the novelty suppressed feeding test (NSFT), light-dark box (LDB) and elevated plus maze (EPM) tests, respectively. In addition, real-time quantitative polymerase chain reaction (qPCR) experiments were carried out to evaluate specific changes in the gene expression of targets involved in stress response [hypothalamus-pituitary-adrenal (HPA) axis] and pharmacological actions of CBD [cannabinoid receptors 1 (CB1r) and 2 (CB2r)], and STR [5-hydroxytryptamine transporter (5HTT)].

## Materials and Methods

### Animals

A total of 94 C57BL/6J male 4-weeks old mice were purchased from Charles River laboratories (Lille, France). Mice, weighed 20–25 g, housed in groups of five per cage (40 × 25 × 22 cm) under controlled environmental conditions (temperature, 23 ± 2°C; relative humidity, 60 ± 10%, and 12 h light/dark cycle, lights on from 08:00 to 20:00 h), in an enriched environment with nesting material and *ad libitum* access to food (Teklad global 18% protein diet, Ref. 2014S, Envigo, Barcelona, Spain), and water except during behavioral evaluation. Experimental procedures were carried out in the animal facilities of Miguel Hernandez University located in San Juan de Alicante (Alicante. Spain). Behavioral evaluation was initiated during the adolescent period of mice (4 weeks old), after one-week acclimatization period to the animal housing room. Experiments were performed during the light cycle (from 16:00 to 18:00 h) placing home cages in the operant-task room 1 h before to start. All experimental procedures complied with the Spanish Royal Decree 53/2013, the Spanish Law 32/2007 and the European Union Directive of the 22nd of September 2010 (2010/63/UE) regulating the care of experimental animals and were approved by the Ethics Committee of Miguel Hernandez University. Animal studies are reported in compliance with the ARRIVE guidelines (Kilkenny et al., 2010; McGrath and Lilley, 2015).

### Drugs

CBD was obtained from STI Pharmaceuticals (Essex, United Kingdom) and was dissolved in ethanol:cremophor:saline (1:1:18) to obtain the required dose of 20 mg/kg for its intraperitoneal administration (i.p.). STR was purchased from Pfizer laboratories (Madrid, Spain) and was dissolved in water to obtain the required dose of 10 mg/kg for its oral administration (p.o.). CBD and STR were freshly prepared every day immediately before its administration at a final volume of 10 ml/kg. Once-daily administration of CBD, STR, CBD plus STR or the corresponding vehicles (from 15:00 to 17:00 h) was carried out between weeks 11 and 14 of the model. A latency time of 90 (CBD) and/or 60 (STR) minutes was left before any behavioral evaluation according to previously published pharmacokinetics data (Deiana et al., 2012; Melis et al., 2012). Drug doses were selected according to prior literature (Wang et al., 2006; Blessing et al., 2015) and to preliminary results obtained with CBD in our laboratory (data not shown).

### Animal Model of Post-Traumatic Stress Disorder

The animal model of PTSD was induced by exposing mice to the following stressful stimuli at different time point for 5 weeks: 1) Fox urine: a perforated plastic tube (50 ml) containing a gauze impregnated in fox urine (Code blue, Fox Urine Cover Scent, Ref. OA1105, 3 ml) or saline (control mice) was placed in the central zone of each cage for 15 min, 2) Unescapable electric shock: animals were placed inside a 50 × 25 × 25 cm acrylic box with a floor consisting of a grid of parallel stainless steel bars (1 mm in diameter and 1 cm apart). Thirty seconds after animals were introduced in the box, they received a 1 mA scrambling shock or not (control mice) during 10 s, with an additional resting time of 20 s, 3) movement restriction: animals were introduced in perforated plastic falcon tubes (50 ml) for 15 min, or were left undisturbed in the home cage (control mice), 4) tilted cage: during dark cycle, home cages were tilted 30° for 12–14 h or not (control mice), 5) wet bedding: during dark cycle, mice were exposed to a cage with wet sawdust bedding for 12–14 h, or were left undisturbed (control mice); and 6) food restriction: during dark cycle, mice were food deprived for 12–14 h, or were left undisturbed (control mice).

**FIGURE 1:**
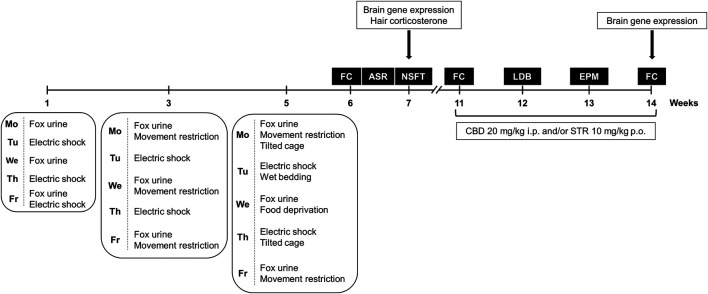
Timeline diagram of the experimental procedure used for the development of the animal model of post-traumatic stress disorder (PTSD) (weeks 1–5) to evaluate PTSD-induced behavioral and neurobiological alterations under basal conditions (weeks 6 and 7), and to analyze the pharmacological actions of cannabidiol (CBD, 20 mg/kg, i.p.) and/or sertraline (STR, 10 mg/kg, p.o.) on long-lasting PTSD disturbances (weeks 11–14). FC: Fear Conditioning, NSFT: Novelty Suppressed Feeding Test, LDB: Light-Dark Box, EPM: Elevated Plus Maze.

Stressful stimuli were applied alternating 3 weeks of exposure (weeks 1, 3, and 5) with two intermediate weeks of resting (weeks 2 and 4), to avoid habituation and to add elements of unpredictability and re-exposure to the stressor. Importantly, the intensity of stress exposure was increased by adding new stressful stimuli from week to week as displayed in the Figure 1. Overall, these experimental aspects are especially relevant to induce long-lasting behavioral and neurochemical alterations in an animal model of PTSD.

### Experimental Design

#### Procedure 1: Evaluation of Basal Behavioral and Neurobiological Alterations Induced by the Animal Model of Post-Traumatic Stress Disorder

This experimental phase was intended to evaluate basal behavioral and neurobiological disturbances induced by the animal model of PTSD (Figure 1). For that purpose, a total of 16 mice were used in this experiment, eight exposed to the animal model of PTSD and eight non-exposed. Fear-related memory and anxiety-like behavior were evaluated at weeks 6 and 7 by the fear conditioning (FC), the acoustic startle response (ASR), and the novelty-suppressed feeding test (NSFT) paradigms. Immediately after the last behavioral evaluation by NSFT mice were killed by cervical dislocation and brain and hair samples were obtained. Brain samples were used for relative gene expression analyses of targets of interest. Hair samples were used for hair accumulated corticosterone quantification as a peripheral biomarker of long-term HPA axis activity. All the behavioral paradigms of this procedure were made under blind conditions.

#### Procedure 2: Evaluation of the Effects of Cannabidiol and/or Sertraline Administration on Long-Lasting Behavioral and Gene Expression Alterations Induced by the Animal Model of Post-Traumatic Stress Disorder

This experimental phase evaluates the effects of CBD and/or STR administration on long-lasting behavioral and gene expression alterations induced by the animal model of PTSD (Figure 1). A total of 78 mice were used, 39 exposed to the animal model of PTSD and 39 non-exposed. After the model induction period and the subsequent basal behavioral evaluations at weeks 6 (FC) and 7 (NSFT), mice were left undisturbed for 3 weeks. After this period, mice were randomly assigned to different treatment groups where CBD and/or STR effects on fear-related memory (weeks 11 and 14) and anxiety-like behavior (weeks 12 and 13) were analyzed. The first administration of CBD and/or STR was carried out 60 and/or 90 min before the FC at week 11, respectively, to evaluate the acute pharmacological effects. Subsequently, both drugs were administered once daily until week 14, evaluating its sub-chronic and chronic effects on different behavioral tests. CBD and/or STR actions on anxiety-like behavior were analyzed by the light-dark box (LDB; week 12) and the elevated plus maze (EPM; week 13) paradigms. At the end of the behavioral evaluation phase, mice were killed by cervical dislocation immediately after the last behavioral test (FC at week 14) and brain samples were removed. These samples were used to analyze relative gene expression of several targets of interest. All the behavioral paradigms of this procedure (FC, NSFT, LDB, and EPM) were made under blind conditions (for more detail see Supplementary Material).

### Behavioral Analyses

#### Fear Conditioning Paradigm

Fear memory retention was evaluated using Pavlovian contextual fear conditioning protocol as described elsewhere (LeDoux, 2000). Briefly, in this behavioral paradigm mice were re-exposed to the same cage where they received electric shocks during the induction of the model of PTSD (or not in the case of control animals), without applying any shock in this evaluation phase. For a total of 5 min, freezing behavior was evaluated as the time of total absence of movements except those necessary to breathe.

#### Acoustic Startle Response

A previously described protocol was used to evaluate acoustic startle response of mice exposed to the animal model of PTSD and controls. Briefly, mice were placed in soundproof chambers equipped with loudspeakers controlled by STARTLE software (Panlab, Barcelona, Spain) (Ortega-Alvaro et al., 2011). Mice movement inside a Plexiglas cylinder was measured by a piezoelectric accelerometer and converted into a digital signal. Mice were acclimatized three days prior to test sessions by placing them each day in the apparatus for 5 min without background noise. The day of the evaluation, mice were exposed to 10 trials of 120 dB (40 ms, 8,000 Hz) acoustic startle stimulus applied every 44 s, recording the maximum of startle amplitude during a 100 ms sampling window.

#### Novelty Suppressed Feeding Test

This behavioral test measures anxiety-induced hyponeophagia as the inhibition of food ingestion or approach to food in an anxiety-provoking environment (Bodnoff et al., 1988; Garcia-Gutierrez et al., 2010). After 24 h of food deprivation, mice were placed in a transparent square cage (40 × 40 × 50 cm) with a single pellet of food left on a white paper platform in the center of the cage. The latency time before the mouse started to eat was recorded up to a threshold period of 5 min. Once the mice started to eat, the total amount of food pellet consumption was measured during an additional 5-minutes time.

#### Light-Dark Box

Anxiety-like behavior was evaluated by the widely accepted LDB paradigm (Crawley and Goodwin, 1980; Garcia-Gutierrez et al., 2018). LDB was carried out in an apparatus with two methacrylate compartments (20 × 20 × 15 cm), one transparent and the other black and opaque, separated by an opaque tunnel (4 cm). Light compartment is illuminated with a lamp (60 W) that is placed 25 cm above it. At the beginning of the 5-min session, mice were placed in the light box facing the tunnel. The total time spent in the light box and the number of transitions between boxes were recorded. A mouse whose four paws were inside the new box was considered as having changed boxes.

#### Elevated Plus Maze

Another commonly used method for evaluating anxiety-like behavior in mice is the EPM (Lister, 1987; Garcia-Gutierrez et al., 2018). The apparatus consists of four arms (two open and two enclosed), that form a plus shape at 50 cm above the floor. The junction of the four arms is a central square platform (5 × 5 cm). At the beginning of the test, mice were placed in the central square, facing one of the enclosed arms. During a period of 5 min, the total time spent in the open arms (calculated as a percentage) and the number of transitions between open and enclosed arms were recorded. Animal arm entry was considered as the entry of its four paws into the arm.

### Gene Expression Studies by Real Time PCR

Relative gene expression of corticotropin releasing factor (*Crf*) in the paraventricular nucleus (PVN), proopiomelanocortin (*Pomc*) in the arcuate nucleus (ARC), glucocorticoid receptor (*GCr*) in the hippocampus (HIPP), *Cnr1*, and *Cnr2* in the amygdala (AMY), and *Slc6a4* in the dorsal raphe nucleus (DR) were analyzed on brain samples obtained in Procedure 1 (week 7) and Procedure 2 (week 14). Briefly, mice were killed at the end of the experimental procedures by cervical dislocation and brains were removed from the skull and frozen at −80°C. Brain sections were cut (500 μm) in a cryostat (−10°C) containing the regions of interest according to Paxinos and Franklin atlas (Paxinos and Franklin, 2001), mounted in the slides and stored at −80°C. Sections were microdissected following the method described by Palkovits and previously performed by our group (Palkovits, 1983; Navarrete et al., 2012). Total RNA was extracted from brain micropunches with TRI Reagent extraction reagent (Applied Biosystem, Madrid, Spain) and reverse transcription was carried out (Applied Biosystem, Madrid, Spain). Quantitative analyses of the relative expression of *Crf* (Mm01293920_s1), *Pomc* (Mm00435874_m1), *GCr* (Mm00433832_m1), *Cnr1* (Mm00432621_s1), *Cnr2* (Mm00438286_m1), and *Slc6a4* (Mm0043939_m1) genes was performed on the StepOne Sequence Detector System (Applied Biosystems, Madrid, Spain). All the reagents used in the study were obtained from Life Technologies, and the manufacturer’s protocols were followed. The reference gene used was 18S rRNA (Mm03928990_g1). Data for each target gene were normalized to the endogenous reference gene, and the fold change in target gene expression was determined using the 2-ΔΔCt method (Livak and Schmittgen, 2001).

### Hair Corticosterone Analysis

After cervical dislocation at week 7 (Procedure 1), mice hair of the dorsal zone was shaved using an electric razor. Hair samples were stored in 1.5 ml polypropylene tubes at -20°C. Extraction and analysis of corticosterone concentration were performed according to a previously described protocol (Erickson et al., 2017). Briefly, hair samples were washed with methanol (5 ml) twice rotating for 3 min. After methanol decantation, samples were placed on aluminum foil and dried in a protected hood for 3 days. Dried samples were weighed and transferred to 2 ml polypropylene tubes containing stainless steel grinding beads (2.8 mm Stainless Steel Grinding Balls Pre-Filled Tubes, OPS Diagnostics, Lebanon, NJ) that were placed in a bead beater (Mixermill MM300, Miguel Hernandez University, Alicante, Spain) to produce a powder. Powdered hair samples were incubated with 1.5 ml of methanol for 24 h on slow rotation to extract steroids. Tubes were centrifuged and steroid-containing supernatants were dried in a protected hood for 2–3 days to evaporate methanol. Dry extracts were analysed by a commercial competitive enzyme-linked immunosorbent assay (ELISA) kit (EIACOR, Invitrogen, Spain) following manufacturer instructions.

### Statistical Analyses

Statistical analyses were performed using Student’s *t*-test for comparing two groups, and two-way analysis of variance (ANOVA) followed by the Student–Newman–Keuls post-hoc test for comparing four groups affected by two variables (treatment with CBD or STR). Differences were considered significant if the probability of error was less than 5%. SigmaPlot 11 software (Systat software Inc., Chicago, IL, United States) was used for all statistical analyses.

## Results

### Procedure 1: Evaluation of Basal Behavioral and Neurobiological Alterations Induced by the Animal Model of Post-Traumatic Stress Disorder

**FIGURE 2:**
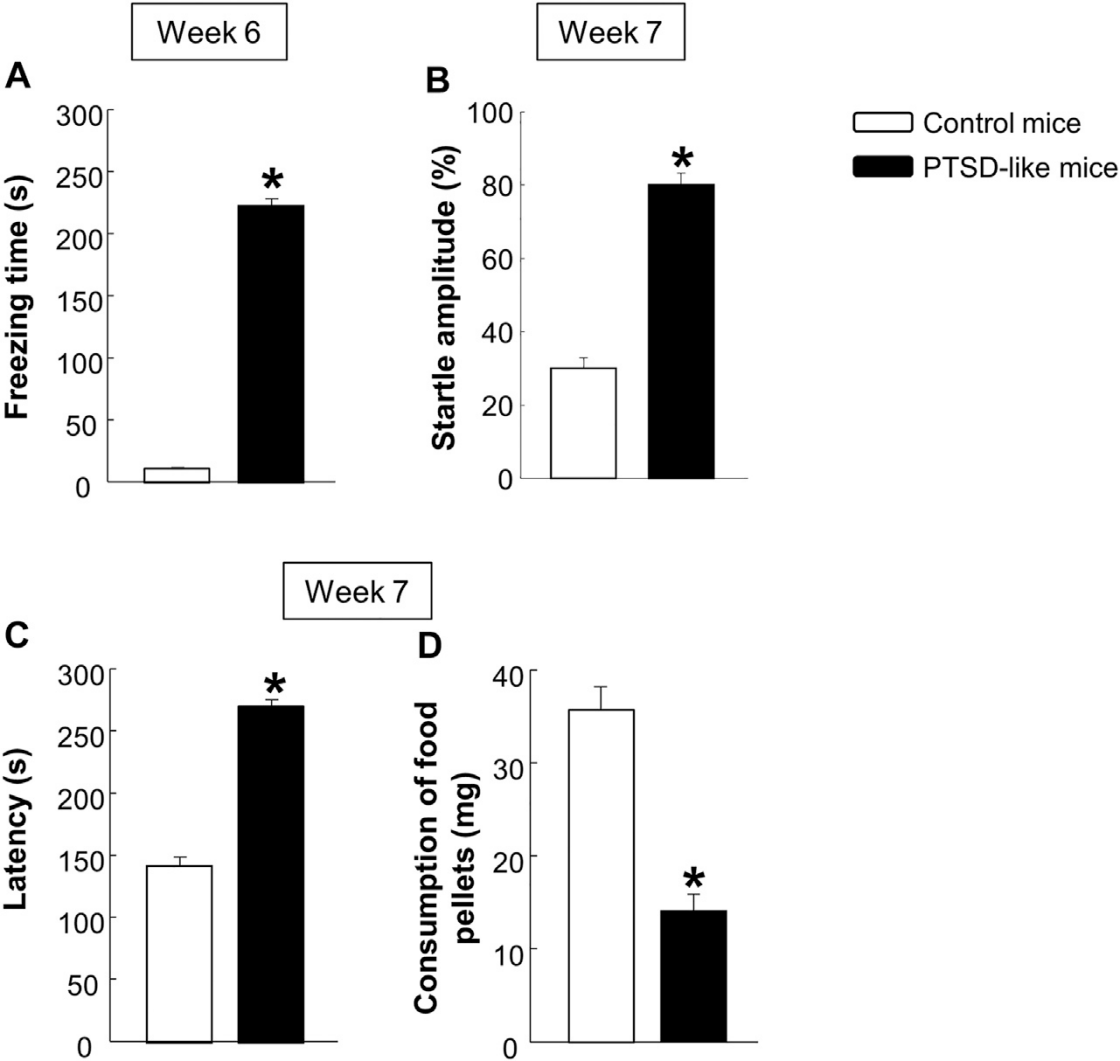
Evaluation of the basal behavioral disturbances induced by the animal model of PTSD at weeks 6 and 7. Analysis of the freezing time (s) by the fear conditioning (FC) paradigm **(A)**, the startle amplitude **(B)** by the acoustic startle response (ASR), and of the latency time **(C)** and food pellets consumption **(D)** by the novelty suppressed feeding test (NSFT). Columns represent the mean and vertical lines ± SEM. *, Values from PTSD-like mice that are significantly different from control mice (Student’s *t*-test, *p* < 0.001). Mice exposed to the PTSD-like model: N = 8; control mice: N = 8.

*Behavioral evaluation*. Mice exposed to the new animal model of PTSD showed a significant increased freezing time in the FC (Figure 2A, Student’s *t*-test, *t* = −13.738, *p* < 0.001, 14 d.f.), enhanced startle response in the ASR (Figure 2B, Student’s *t*-test, *t* = −3.002, *p* < 0.01, 14 d.f.), and increased latency time (Figure 2B, Student’s *t*-test, *t* = −6.824, *p* < 0.001, 14 d.f.) with decreased food consumption (Figure 2C, Student’s *t*-test, *t* = 2.202, *p* < 0.05, 14 d.f.) in the NSFT, in comparison with control mice. According to these basal behavioral results, mice were randomly assigned to four experimental groups to be treated with CBD and/or STR or its corresponding vehicle in Procedure 2 (for more detail about mice assignment see Supplementary Figure S1).

**FIGURE 3:**
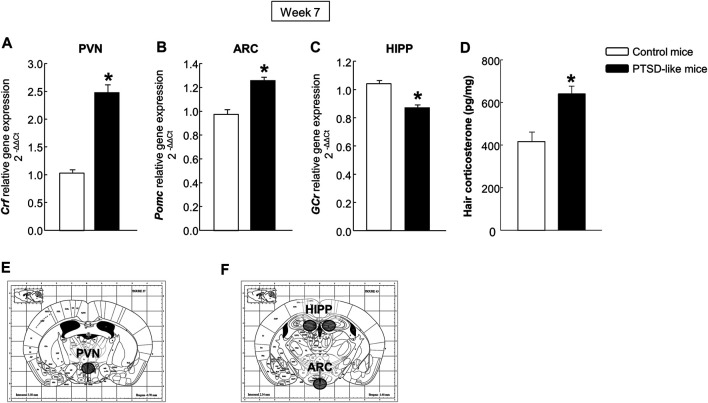
Relative gene expression analyses of corticotropin releasing factor (*Crf*) in the paraventricular nucleus (PVN) **(A)**, proopiomelanocortin (*Pomc*) in the arcuate nucleus (ARC) **(B)**, and glucocorticoid receptor (*GCr*) in the hippocampus (HIPP) **(C)** by real time PCR. Quantification of hair corticosterone (pg/mg) **(D)** by Enzyme-Linked ImmunoSorbent Assay (ELISA). Columns represent the mean and vertical lines ± SEM of 2^-∆∆C^. *, Values from PTSD-like mice that are significantly different from control mice (Student’s *t*-test, *p* < 0.001). Mice exposed to the PTSD-like model: N = 8; control mice: N = 8. **(E,F)** Representative images from *Paxinos and Franklin*’*s mouse brain atlas* including the selected coronal sections to microdissect the regions of interest.

*Gene expression analyses*. Statistical analyses indicated increased *Crf* (Figure 3A, Student’s *t*-test, *t* = −9.349, *p* < 0.001, 14 d.f.) and *Pomc* (Figure 3B, Student’s *t*-test, *t* = −5.565, *p* < 0.001, 14 d.f.) relative gene expression levels in the PVN and ARC, respectively, and decreased gene expression of *GCr* (Figure 3C, Student’s *t*-test, *t* = 5.734, *p* < 0.001, 14 d.f.) in the HIPP of PTSD-like mice compared with control mice. These changes were accompanied by an increased corticosterone concentration in mice hair compared with controls (Figure 3D, Student’s *t*-test, *t* = −3.943, *p* < 0.01, 14 d.f.).

**FIGURE 4:**
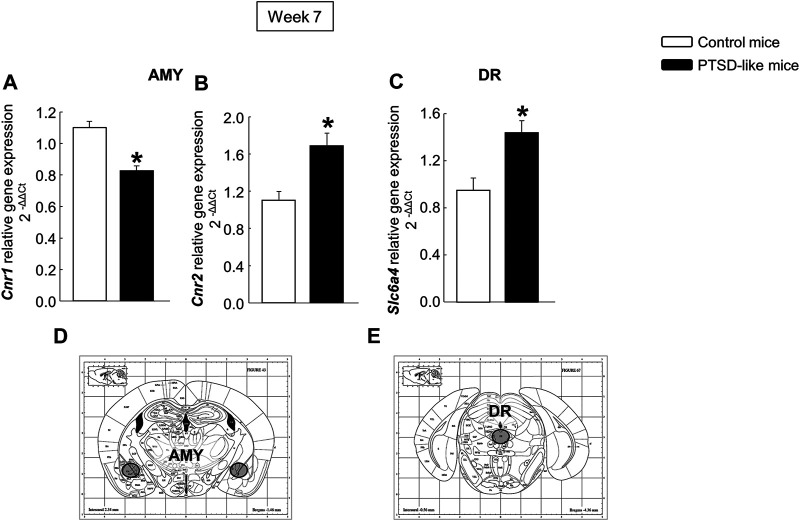
Relative gene expression analyses of cannabinoid receptors 1 (*Cnr1*) **(A)** and 2 (*Cnr2*) **(B)** in the amygdala (AMY), and serotonin transporter (*Slc6a4*) in the dorsal raphe nucleus (DR) **(C)** by real time PCR. Columns represent the mean and vertical lines ± SEM of 2^−∆∆C^. *, Values from PTSD-like mice that are significantly different from control mice (Student’s *t*-test, *p* < 0.001). Mice exposed to the PTSD-like model: N = 8; control mice: N = 8. **(D,E)** Representative images from *Paxinos and Franklin*’*s mouse brain atlas* including the selected coronal sections to microdissect the regions of interest.

In addition, mice exposed to the animal model of PTSD showed reduced *Cnr1* (Figure 4A, Student’s *t*-test, *t* = 5.647, *p* < 0.001, 14 d.f.) and increased *Cnr2* (Figure 4B, Student’s *t*-test, *t* = −3.604, *p* = 0.003, 14 d.f.) gene expression in the AMY, as well as enhanced gene expression of *Slc6a4* (Figure 4C, Student’s *t*-test, *t* = −3.337, *p* = 0.005, 14 d.f.) in the DR compared with non-exposed mice.

### Procedure 2: Evaluation of the Effects of Cannabidiol and/or Sertraline on Long-Lasting Behavioral and Gene Expression Alterations Induced by the Animal Model of Post-Traumatic Stress Disorder

#### Effects of Cannabidiol and/or Sertraline on Fear-Related Memory and Anxiety-Like Behavior Disturbances Induced by the Animal Model of Post-Traumatic Stress Disorder

**FIGURE 5:**
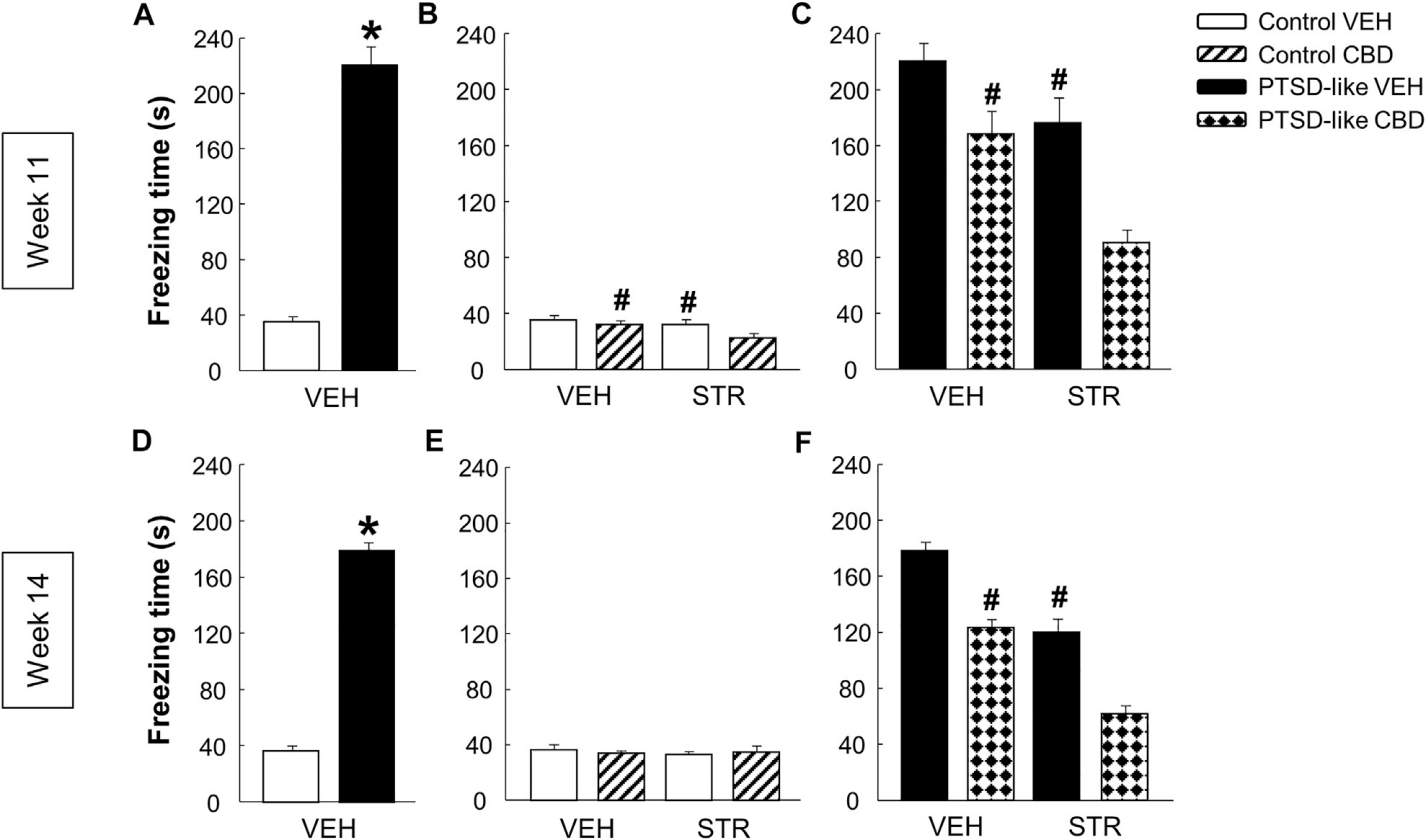
Evaluation of fear-related memory at weeks 11 and 14. Analysis of the freezing time (s) by the fear conditioning paradigm between control and PTSD-like VEH-treated mice at week 11 **(A)** and week 14 **(D)**. Effects of acute (week 11) and chronic (week 14) administration of CBD (20 mg/kg, i.p.) and/or STR (10 mg/kg, p.o.), or its corresponding VEH, on the freezing time (s) of control mice **(B, E)** and PTSD-like mice **(C,F)**. Columns represent the means and vertical lines ± SEM. *, Values from PTSD-like VEH-treated mice that are significantly different from control VEH-treated mice (Student’s *t*-test, *p* < 0.001). #, Values from CBD- or STR-treated groups that are significantly different from VEH-treated mice (Two-way ANOVA, *p* < 0.05). and, Values from CBD plus STR-treated mice that significantly different from CBD- and STR-treated control (Two-way ANOVA, *p* < 0.05) and PTSD-like mice (Two-way ANOVA, *p* < 0.001). Mice exposed to the PTSD-like model: N(VEH) = 10, N(CBD) = 10, N(STR) = 9, N(CBD plus STR) = 10; control mice: N(VEH) = 10, N(CBD) = 9, N(STR) = 9, N(CBD plus STR) = 10.

*Fear conditioning paradigm.* Statistical analyses revealed a higher mean freezing time in PTSD-like mice compared with control mice at week 11 (Figure 5A, Student’s *t*-test, *t* = −14.178, *p* < 0.001, 18 d.f.) and week 14 (Figure 5D, Student’s *t*-test, *t* = −21.269, *p* < 0.001, 18 d.f.). Within control group, no significant differences were observed between CBD plus STR-treated animals compared to CBD and STR-treated mice at week 11 (Figure 5B, Two-way ANOVA, CBD: F_(1,37)_ = 4.794, *p* < 0.05; STR: F_(1,37)_ = 4.712, *p* < 0.05; CBD x STR: F_(1,37)_ = 1.140, *p* = 0.293), and at week 14 (Figure 5E, Two-way ANOVA, CBD: F_(1,37)_ = 0.006, *p* = 0.940; STR: F_(1,37)_ = 0.201, *p* = 0.657; CBD x STR: F_(1,37)_ = 0.456, *p* = 0.504). In PTSD-like mice, CBD and STR treatments significantly reduced the freezing time at week 11 (acute treatment), reaching a more pronounced reduction at week 14 (repeated treatment). Interestingly, pharmacological combination of CBD plus STR, compared with CBD or STR alone, achieved a superior effect in the reduction of the freezing time in mice exposed to the PTSD model (without reaching statistical significance) at week 11 (Figure 5C, Two-way ANOVA, CBD: F_(1,38)_ = 24.661, *p* < 0.001; STR: F_(1,38)_ = 19.226, *p* < 0.001; CBD x STR: F_(1,38)_ = 1.488, *p* = 0.231) and at week 14 (Figure 5F, Two-way ANOVA, CBD: F_(1,38)_ = 76.676, *p* < 0.001; STR: F_(1,38)_ = 86.029, *p* < 0.001; CBD x STR: F_(1,38)_ = 0.0823, *p* = 0.776).

**FIGURE 6:**
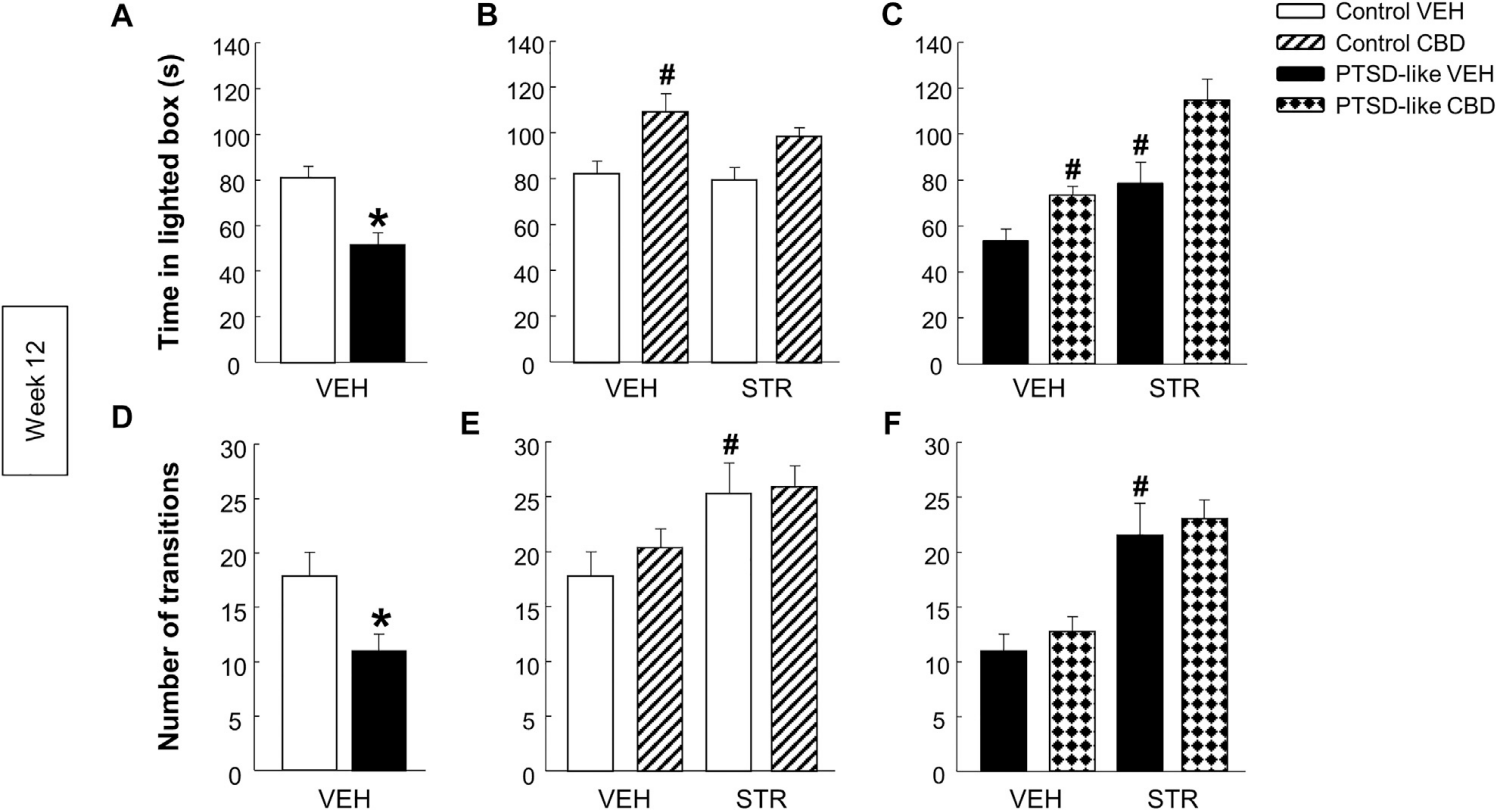
Evaluation of anxiety-like behavior by the light-dark box (LDB) paradigm at week 12. Comparative analysis between control and PTSD-like VEH-treated mice of the time spent in the lighted box (s) **(A)** and the number of transitions **(D)**. Effects of CBD (20 mg/kg, i.p.) and/or STR (10 mg/kg, p.o.) administration, or its corresponding VEH, on the time spent in the lighted box (s) **(B,C)** and the number of transitions **(E,F)** of control and PTSD-like mice. Columns represent the mean and vertical lines ± SEM. *, Values from PTSD-like VEH-treated mice that are significantly different from control VEH-treated mice (Student’s *t*-test, *p* < 0.001). #, Values from CBD- or STR-treated groups that are significantly different from VEH-treated mice (Two-way ANOVA, *p* < 0.001). and, Values from CBD plus STR-treated mice that are significantly different from CBD- and STR-treated mice (Two-way ANOVA, *p* < 0.001). Mice exposed to the PTSD-like model: N(VEH) = 10, N(CBD) = 10, N(STR) = 9, N(CBD plus STR) = 10; control mice: N(VEH) = 10, N(CBD) = 9, N(STR) = 9, N(CBD plus STR) = 10.

*Light-dark box.* PTSD-like mice spent less time in the lighted box (Figure 6A, Student’s *t*-test, *t* = 4.190, *p* < 0.001, 18 d.f.) than control mice. Additionally, the number of transitions was reduced in PTSD-like mice compared with control mice (Figure 6D, Student’s *t*-test, *t* = 2.535, *p* < 0.05, 18 d.f.). Within control mice group, only CBD treatment significantly increased the time spent in the lighted box (Figure 6B, Two-way ANOVA, CBD: F_(1,37)_ = 16.739, *p* < 0.001; STR: F_(1,37)_ = 1.400, *p* = 0.245; CBD x STR: F_(1,37)_ = 0.508, *p* = 0.481). Within PTSD-like mice, both CBD and STR treatment increased the time spent in the lighted box. Interestingly, CBD plus STR combination increased the time of permanence in the lighted box compared with CBD or STR alone, without reaching statistical significance (Figure 6C, Two-way ANOVA, CBD: F_(1,38)_ = 16.271, *p* < 0.001; STR: F_(1,38)_ = 22.939, *p* < 0.001; CBD x STR: F_(1,38)_ = 1.394, *p* = 0.246). STR treatment increased the number of transitions in both control and PTSD-like mice (Figure 6E, Two-way ANOVA, CBD: F_(1,37)_ = 0.593, *p* = 0.446; STR: F_(1,37)_ = 9.272, *p* < 0.01; CBD x STR: F_(1,37)_ = 0.212, *p* = 0.648; and Figure 6F, Two-way ANOVA, CBD: F_(1,38)_ = 0.774, *p* = 0.385; STR: F_(1,38)_ = 30.088, *p* < 0.001; CBD x STR: F_(1,38)_ = 0.00452, *p* = 0.947).

**FIGURE 7:**
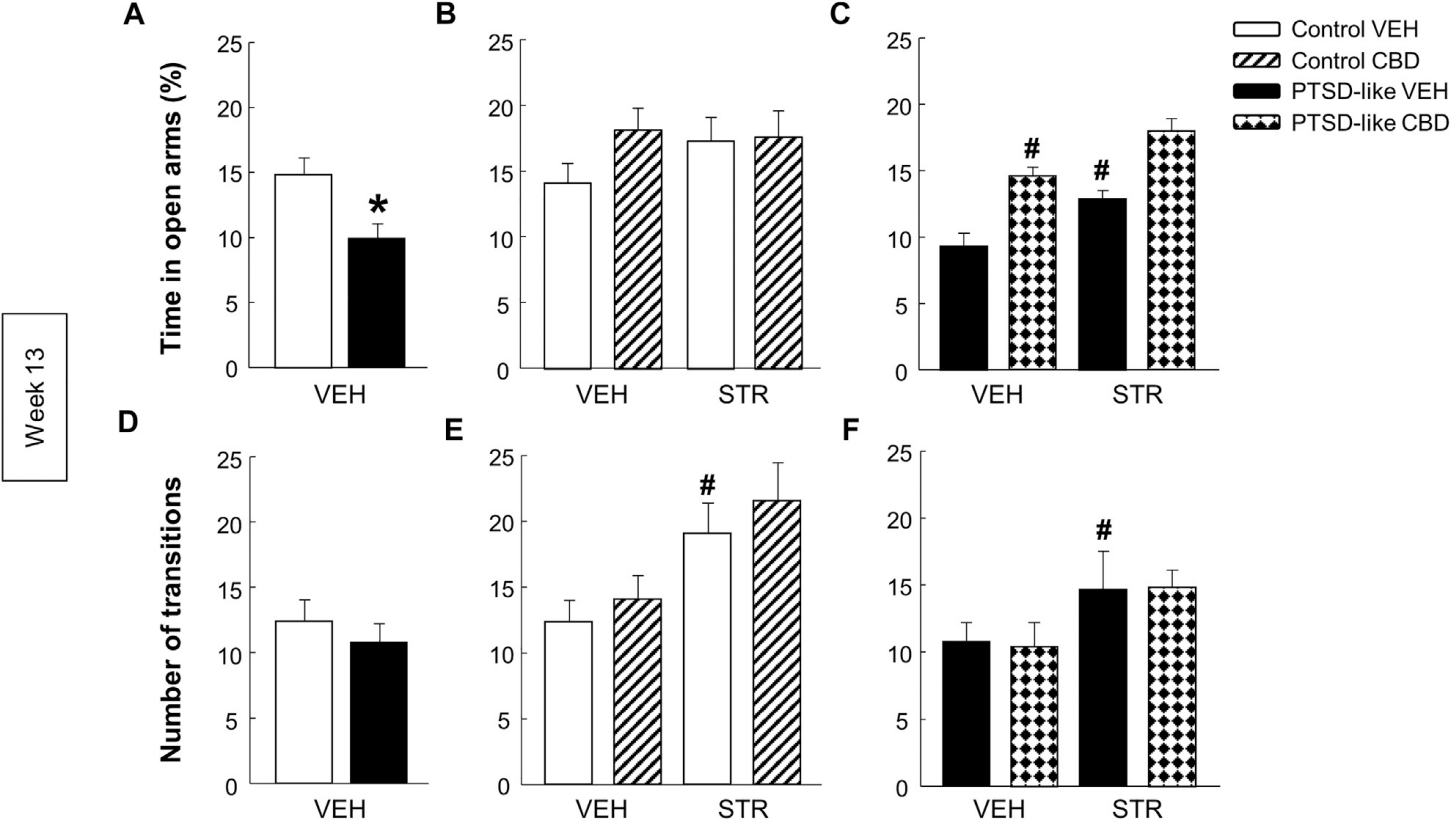
Evaluation of anxiety-like behavior by the elevated plus maze (EPM) paradigm at week 13. Comparative analysis between control and PTSD-like VEH-treated mice of the time spent in the open arms (s) **(A)** and the number of transitions **(D)**. Effects of CBD (20 mg/kg, i.p.) and/or STR (10 mg/kg, p.o.) administration, or its corresponding VEH, on the time spent in the open arms (s) **(B,C)** and the number of transitions **(E,F)** of control and PTSD-like mice. Columns represent the mean and vertical lines ± SEM. *, Values from PTSD-like VEH-treated mice that are significantly different from control VEH-treated mice (Student’s *t*-test, *p* < 0.001). #, Values from CBD- or STR-treated groups that are significantly different from VEH-treated mice (Two-way ANOVA, *p* < 0.001). and, Values from CBD plus STR-treated mice that are significantly different from CBD- and STR-treated mice (Two-way ANOVA, *p* < 0.001). Mice exposed to the PTSD-like model: N(VEH) = 10, N(CBD) = 10, N(STR) = 9, N(CBD plus STR) = 10; control mice: N(VEH) = 10, N(CBD) = 9, N(STR) = 9, N(CBD plus STR) = 10.

*Elevated plus maze.* PTSD-like mice spent less time in the open arms than control mice (Figure 7A, Student’s *t*-test, *t* = 2.962, *p* < 0.01, 18 d.f.), and no differences were observed in the number of transitions between opened and closed arms (Figure 7D, Student’s *t*-test, *t* = 0.750, *p* = 0.463, 18 d.f.). Within control mice no differences were observed in the time spent in open arms (Figure 6B, Two-way ANOVA, CBD: F_(1,37)_ = 1.564, *p* = 0.220; STR: F_(1,37)_ = 0.584, *p* = 0.450; CBD x STR: F_(1,37)_ = 1.202, *p* = 0.281). However, within PTSD-like mice, CBD or STR treatment significantly increased the time spent in the open arms, effect that was more pronounced with the CBD plus STR combination without reaching statistical significance (Figure 7C, Two-way ANOVA, CBD: F_(1,38)_ = 41.191, *p* < 0.001; STR: F_(1,38)_ = 18.328, *p* < 0.001; CBD x STR: F_(1,38)_ = 0.008, *p* = 0.927). STR treatment increased the number of transitions in control mice (Figure 7E, Two-way ANOVA, CBD: F_(1,37)_ = 0.923, *p* = 0.343; STR: F(1,37) = 10.553, *p* < 0.01; CBD x STR: F_(1,37)_ = 0.0317, *p* = 0.860) and in PTSD-like mice (Figure 7F, Two-way ANOVA, CBD: F_(1,38)_ = 0.000317, *p* = 0.986; STR: F_(1,38)_ = 4.869, *p* < 0.05; CBD x STR: F_(1,38)_ = 0.0203, *p* = 0.888).

#### Effects of Cannabidiol and/or Sertraline on Relative Gene Expression Alterations Induced by the Animal Model of Post-Traumatic Stress Disorder

**FIGURE 8:**
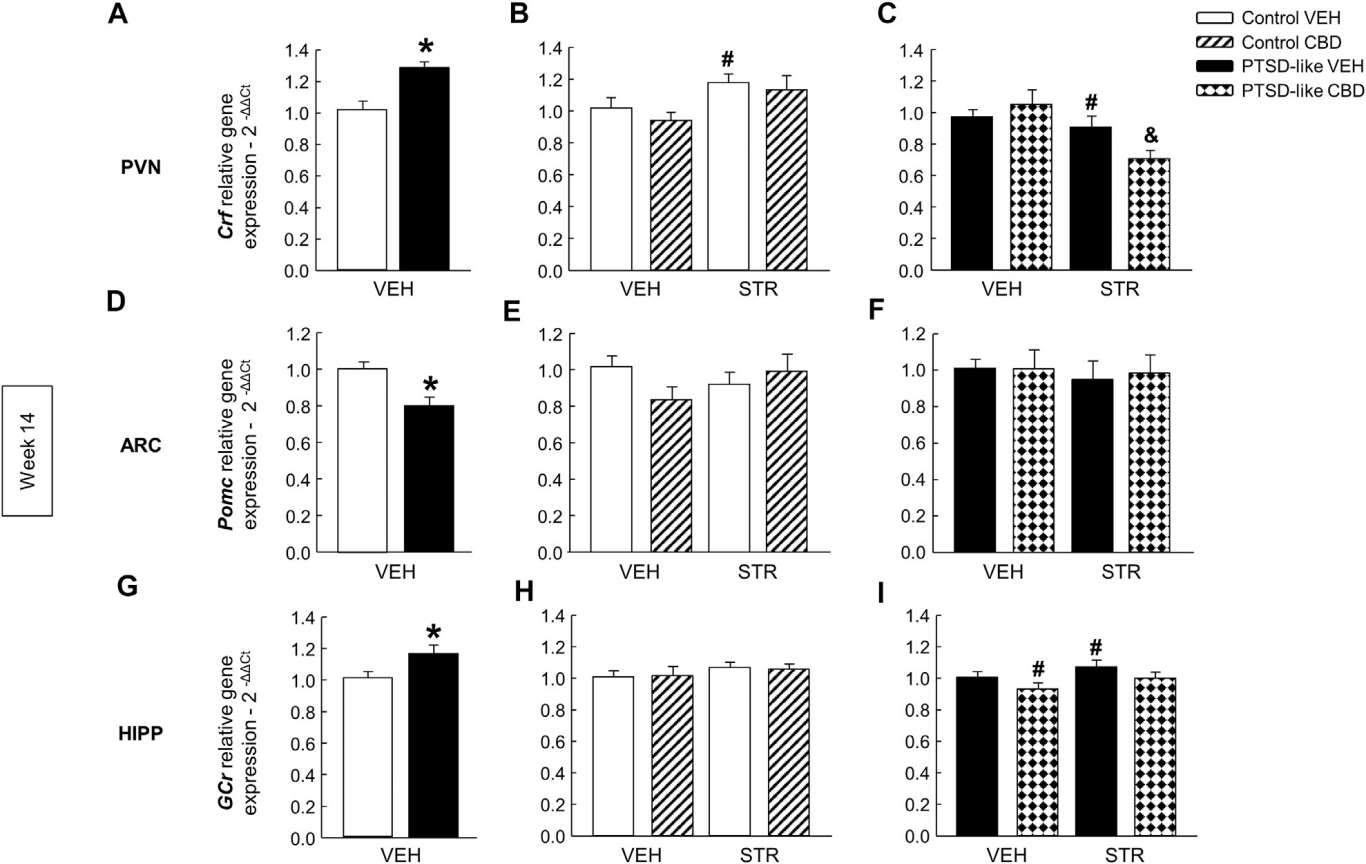
Relative gene expression analyses of Hypothalamus-Pituitary-Adrenal (HPA) axis markers by real time PCR at week 14. Comparative analysis between control and PTSD-like VEH-treated mice of the relative gene expression of corticotropin releasing factor (*Crf*) in the paraventricular nucleus (PVN) **(A)**, proopiomelanocortin (*Pomc*) in the arcuate nucleus (ARC) **(D)**, and glucocorticoid receptor (*GCr*) in the hippocampus **(G)**. Effects of CBD (20 mg/kg, i.p.) and/or STR (10 mg/kg, p.o.) administration, or its corresponding VEH, on the relative gene expression of *Crf* in the PVN **(B,C)**, *Pomc* in the ARC **(E,F)**, and *GCr* in the HIPP **(H,I)** of control and PTSD-like mice. Columns represent the means and vertical lines ± SEM of 2^-∆∆Ct^. *, Values from PTSD-like mice that are significantly different from control mice (Student’s *t*-test, *p* < 0.05). #, Values from CBD- or STR-treated mice that are significantly different from VEH-treated mice (Two-way ANOVA, *p* < 0.05). and, Values from CBD plus STR-treated mice that are significantly different from CBD- and STR-treated mice (Two-way ANOVA, *p* < 0.05). Mice exposed to the PTSD-like model: N(VEH) = 10, N(CBD) = 9, N(STR) = 9, N(CBD plus STR) = 10; control mice: N(VEH) = 10, N(CBD) = 9, N(STR) = 9, N(CBD plus STR) = 10.

*HPA axis*. *Crf* gene expression increased in the PVN of PTSD-like mice compared with control mice (Figure 8A, Student’s *t*-test, *t* = −3.459, *p* < 0.01, 18 d.f.). Within the control group, STR treatment induced an upregulation in *Crf* gene expression (Figure 8B, Two-way ANOVA; CBD: F_(1,37)_ = 0.861, *p* = 0.360; STR: F_(1,37)_ = 6.702, *p* < 0.05; CBD x STR: F_(1,37)_ = 0.0597, *p* = 0.808). In the PTSD-like mice group, Two-way ANOVA revealed that STR reduced the gene expression of *Crf*, achieving a more pronounced reduction when combined with CBD (Figure 8C, Two-way ANOVA, CBD: F_(1,38)_ = 0.733, *p* = 0.398; STR: F_(1,38)_ = 8.885, *p* < 0.01; CBD x STR: F_(1,38)_ = 4.246, *p* < 0.05). In addition, PTSD-exposed mice also showed decreased gene expression of *Pomc* in the ARC compared with control mice (Figure 8D, Student’s *t*-test, *t* = 3.416, *p* < 0.01, 18 d.f.), but no differences were observed in both control (Figure 8E, Two-way ANOVA, CBD: F_(1,38)_ = 0.561, *p* = 0.459; STR: F_(1,38)_ = 0.158, *p* = 0.693; CBD x STR: F_(1,38)_ = 2.859, *p* = 0.100) and PTSD-like mice (Figure 8F, Two-way ANOVA; CBD: F_(1,37)_ = 0.0340, *p* = 0.855; STR: F_(1,37)_ = 0.233, *p* = 0.632; CBD x STR: F_(1,37)_ = 0.0370, *p* = 0.849) after CBD and/or STR administration. Finally, *GCr* gene expression in the HIPP increased in PTSD-like mice compared with controls (Figure 8G, Student’s *t*-test, *t* = −2.359, *p* < 0.05, 18 d.f.) and no differences were observed among the four groups of control treated mice (Figure 8H, Two-way ANOVA, CBD: F_(1,37)_ = 1.621, *p* = 0.212; STR: F_(1,37)_ = 0.00139, *p* = 0.970; CBD x STR: F_(1,37)_ = 0.0626, *p* = 0.804). Within PTSD-like group, CBD decreased the *GCr* gene expression and STR increased it (Figure 8I, Two-way ANOVA, CBD: F_(1,38)_ = 6.94, *p* < 0.05; STR: F_(1,38)_ = 6.022, *p* < 0.05; CBD x STR: F_(1,38)_ = 0.414, *p* = 0.524).

**FIGURE 9:**
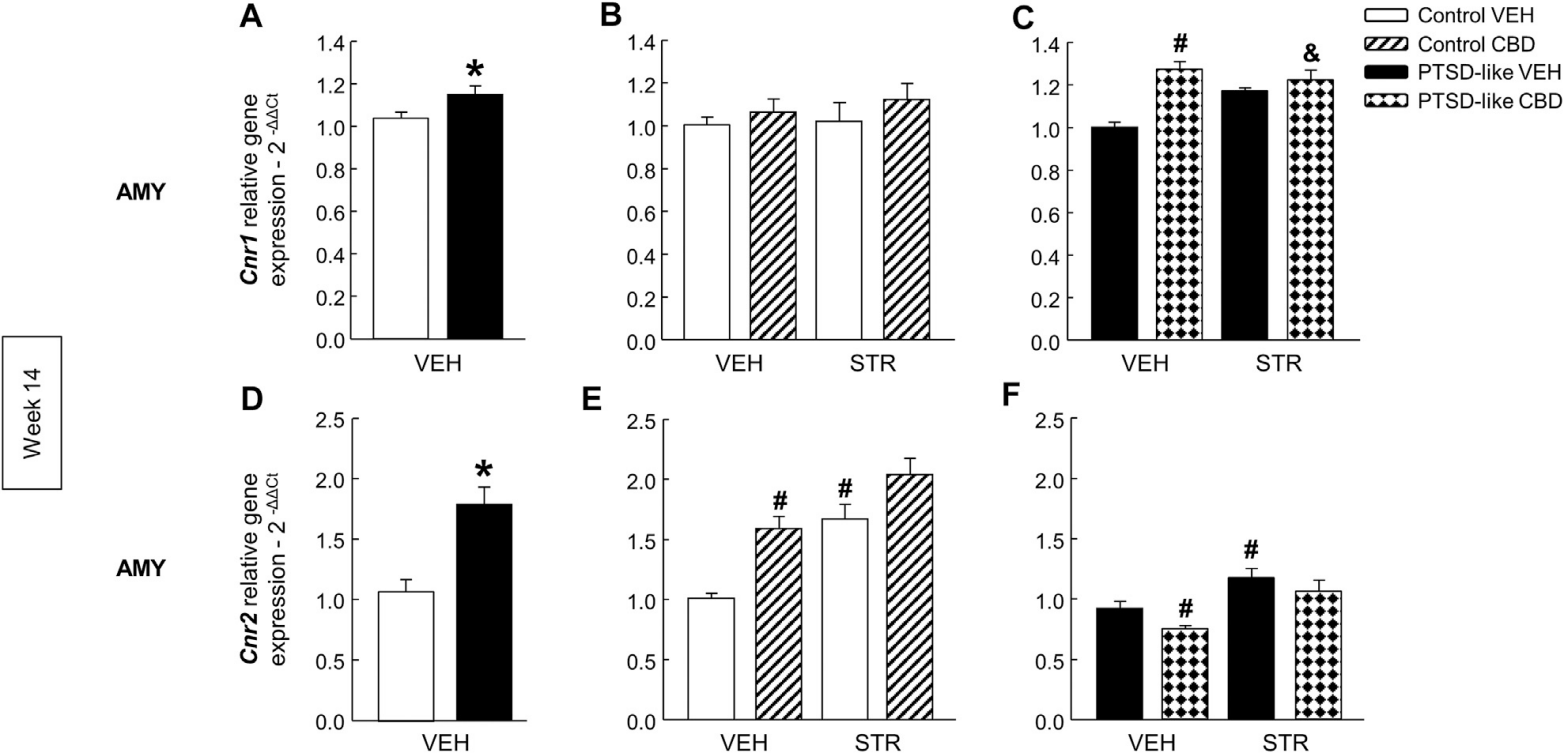
Relative gene expression analyses of cannabinoid receptor 1 (*Cnr1*) and 2 (*Cnr2*) in the amygdala (AMY) by real time PCR at week 14. Comparative analysis between control and PTSD-like vehicle (VEH)-treated mice of the relative gene expression of *Cnr1* **(A)** and *Cnr2* **(D)**. Effects of CBD (20 mg/kg) and/or STR (10 mg/kg) administration, or its corresponding VEH, on the relative gene expression of *Cnr1* **(B,C)** and *Cnr2* **(E,F)** of control and PTSD-like mice. Columns represent the means and vertical lines ± SEM of 2^-∆∆Ct^. *, Values from PTSD-like mice that are significantly different from control mice (Student’s *t*-test, *Cnr1*: *p* < 0.05, *Cnr2*: *p* < 0.001). #, Values from CBD- or STR-treated mice that are significantly different from VEH-treated mice (Two-way ANOVA, *p* < 0.05). and, Values from CBD plus STR-treated mice that are significantly different from CBD- and STR-treated mice (Two-way ANOVA, *p* < 0.05). Mice exposed to the PTSD-like model: N(VEH) = 10, N(CBD) = 9, N(STR) = 9, N(CBD plus STR) = 10; control mice: N(VEH) = 10, N(CBD) = 9, N(STR) = 9, N(CBD plus STR) = 10.

*Cannabinoid receptors*. *Cnr1* gene expression was significantly increased in PTSD-like mice compared with controls (Figure 9A, Student’s *t*-test, *t* = −2.223, *p* < 0.05, 18 d.f.). Within control group, no differences were observed in the *Cnr1* gene expression with drug administration (Figure 9B, Two-way ANOVA, CBD: F_(1,37)_ = 1.421, *p* = 0.241; STR: F_(1,37)_ = 0.319, *p* = 0.576; CBD x STR: F_(1,37)_ = 0.105, *p* = 0.747). Nevertheless, CBD and its combination with STR increased the *Cnr1* gene expression in PTSD-like mice (Figure 9C, Two-way ANOVA, CBD: F_(1,39)_ = 18.716, *p* < 0.001; STR: F_(1,39)_ = 0.583, *p* = 0.450; CBD x STR: F_(1,39)_ = 9.955, *p* < 0.01). In addition, *Cnr2* gene expression was significantly increased in PTSD-like mice (Figure 9D, Student’s *t*-test, *t* = −4.763, *p* < 0.001, 18 d.f.). In the control group, CBD or STR increased *Cnr2* gene expression (Figure 9E, Two-way ANOVA, CBD: F_(1,39)_ = 20.301, *p* < 0.001; STR: F_(1,39)_ = 27.577, *p* < 0.001; CBD x STR: F_(1,39)_ = 1.052, *p* = 0.312). In addition, in the PTSD-like group CBD treatment decreased while STR increased *Cnr2* gene expression (Figure 9F, Two-way ANOVA, CBD: F_(1,39)_ = 4.281, *p* < 0.05; STR: F_(1,39)_ = 17.287, *p* < 0.001; CBD x STR: F_(1,39)_ = 0.179, *p* = 0.675).

**FIGURE 10:**
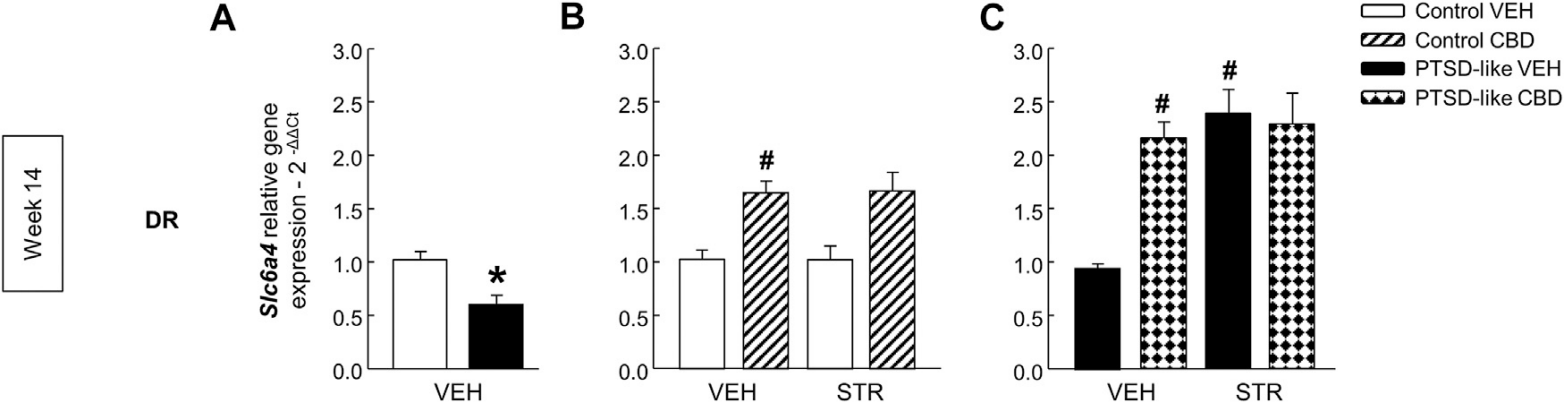
Relative gene expression analyses of serotonin transporter (*Slc6a4*) in the dorsal raphe nucleus (DR) by real time PCR at week 14. Comparative analysis between control and PTSD-like vehicle (VEH)-treated mice of the relative gene expression of *Slc6a4* **(A)** in the DR. Effects of CBD (20 mg/kg) and/or STR (10 mg/kg) administration, or its corresponding VEH, on the relative gene expression of *Slc6a4* in the DR of control **(B)** and PTSD-like **(C)** mice. Columns represent the means and vertical lines ± SEM of 2^-∆∆Ct^. *, Values from PTSD-like mice that are significantly different from control mice (Student’s *t*-test, *Slc6a4*: *p* < 0.001). #, Values from CBD- or STR-treated mice that are significantly different from VEH-treated mice (Two-way ANOVA, *p* < 0.05). Mice exposed to the PTSD-like model: N(VEH) = 10, N(CBD) = 9, N(STR) = 9, N(CBD plus STR) = 10; control mice: N(VEH) = 10, N(CBD) = 9, N(STR) = 9, N(CBD plus STR) = 10.

*Serotonin transporter*. *Slc6a4* gene expression was significantly decreased in PTSD-like mice (Figure 10A, Student’s *t*-test, *t* = 3.550, *p* < 0.01, 18 d.f.). Within control group, only CBD enhanced *Slc6a4* gene expression (Figure 10B, Two-way ANOVA, CBD: F_(1,39)_ = 25.440, *p* < 0.001; STR: F_(1,39)_ = 0.0931, *p* = 0.762; CBD x STR: F_(1,39)_ = 0.113, *p* = 0.739). Within PTSD-like mice, CBD or STR significantly increased *Slc6a4* gene expression in comparison with VEH-treated group, and a similar effect was reached with CBD plus STR combination but without achieving statistical significance (Figure 10C, Two-way ANOVA, CBD: F_(1,38)_ = 9.050, *p* < 0.01; STR: F_(1,38)_ = 10.984, *p* < 0.01; CBD x STR: F_(1,38)_ = 2.726, *p* = 0.108).

## Discussion

The results of the present study reveal that the administration of CBD alone or in combination with STR significantly regulated the long-lasting behavioral and neurochemical disturbances in this animal model of PTSD. This statement is supported by the following observations: 1) Mice exposed to the animal model of PTSD showed a pronounced increase of fear-related memories, hyperarousal and anxiety-like behaviors together with gene expression alterations in the HPA-axis, *Cnr1*, *Cnr2* and *Slc6a4* genes, including higher hair accumulated corticosterone concentrations, 2) Exposure of mice to the animal model of PTSD produced a long-lasting enhancement of fear-related memories and anxiety-like behaviors, as well as gene expression changes in HPA-axis, *Cnr1*, *Cnr2* and *Slc6a4* genes, and 3) The administration of CBD (20 mg/kg, i.p.), STR (10 mg/kg, p.o.) and its combination significantly reduced fear-related memories, anxiety-like behaviors and long-term gene expression alterations of PTSD-like mice.

For improving the understanding of the pathophysiological hallmarks of PTSD, it is crucial the development of animal models to reproduce, at least in part, the intensity and the duration of PTSD symptoms. These are critical to identify therapeutic targets leading to safer and more effective pharmacological strategies. In the present study, a chronic animal model of PTSD was developed to induce intense and long-lasting emotional and brain gene expression disturbances. The development of the animal model of PTSD was carried out during mice adolescent period, being this fact critical to induce pronounced and long-lasting alterations related with the exposure to early traumatic experiences. Indeed, mice exposed to this model showed remarkable and enduring disturbances in fear extinction and anxiety-like behavior, that were notorious even 9 weeks after the end of the induction. In the FC, the small reduction of the freezing time observed at weeks 11 (1.38%) and 14 (20.18%) compared to the week 6 (baseline) highlights the fear extinction deficits in mice exposed to the animal model of PTSD. Therefore, it is worth to mention that the long-term PTSD-related impairments triggered by this model facilitate the simulation, at least in part, of chronic emotional disturbances in PTSD patients, and results ideal to evaluate the effects of chronic drug treatments (that usually require 3–5 weeks to result effective).

In the present study, 2-weeks after the end of the induction of the PTSD model, gene expression analyses revealed that *Crf* and *Pomc* were significantly increased in the PVN and ARC of PTSD animals, respectively. In addition, hair accumulated corticosterone was also elevated, confirming the maintained hyperactivity of the HPA axis during the PTSD model. Furthermore, *GCr* gene expression was downregulated in the HIPP. This effect may be related, at least in part, with the increase of corticosterone concentrations and the negative feed-back regulation. On week 14, we found a moderate increase of *Crf* gene expression in the PVN whereas *Pomc* gene expression was reduced in the ARC and *GCr* gene expression increased in the HIPP. It is tempting to hypothesize that a long-term reduction of HPA axis activity may underlie these alterations, in accordance with the clinical evidence pointing out that PTSD at advanced stages may be associated with hypocortisolism (Miller et al., 2007). However, future studies are needed to further characterize long-term disturbances in the regulation of the HPA axis.

The endogenous cannabinoid and serotonergic systems are strongly involved in the regulation of the emotional response. Mice exposed to the animal model showed reduced gene expression of *Cnr1* in the AMY and enhanced gene expression of *Cnr2* in the AMY and *Slc6a4* in the DR, suggesting the involvement of these targets in the behavioral changes observed in PTSD-like mice under basal conditions (weeks 6 and 7). Interestingly, up-regulation of *Cnr2* gene expression was maintained at week 14, whereas for *Cnr1* and *Slc6a4* an opposite effect was observed in the long-term.

Recently, some preclinical and clinical reports suggested the usefulness of CBD as a new alternative for the treatment of PTSD (Schier et al., 2012; Jurkus et al., 2016; Shannon and Opila-Lehman, 2016; Bitencourt and Takahashi, 2018; Crippa et al., 2018; Bonaccorso et al., 2019; Elms et al., 2019; Lisboa et al., 2019). Despite the available, although scarce, reports in rodents regarding the effects of CBD on fear extinction or anxiety-like behavior in animal models of PTSD (Campos et al., 2012; Shallcross et al., 2019), no previous studies evaluated the effects of the repeated administration of CBD, alone or in combination with STR, on long-term behavioral and neurochemical alterations produced by a chronic animal model of PTSD. The results demonstrate that CBD significantly attenuated freezing time under acute and chronic administration and produced an anxiolytic action on the LDB and EPM paradigms, effects that were similar to those produced by STR. Interestingly, the combination of CBD plus STR induced more pronounced effects, reducing the freezing time approximately by half, and producing a higher increase of the latency time in the lighted box or open arms. Therefore, these results provide novel and relevant information regarding the therapeutic potential of CBD but especially of CBD plus STR combination for attenuating trauma-related memories and anxiety-like behaviors in PTSD.

Real time PCR analyses revealed that the long-lasting increase of *Crf* in the PVN of PTSD-like animals was significantly normalized by CBD plus STR combination whereas no changes were observed in control mice. Furthermore, upregulation of *GCr* in the HIPP of PTSD-like mice was only normalized by CBD while no changes were observed in control animals. These results are related with previous reports by our group showing that low to moderate doses of CBD did not change *Crf*, *Pomc* and *GCr* gene expressions in non-stressed mice whereas an intermediate CBD dose (15 mg/kg) induced a normalization effect in mice exposed to acute restraint stress (Viudez-Martinez et al., 2018).

Furthermore, gene expression analyses of *Cnr1* and *Cnr2* in the AMY, and *Slc6a4* in the DR were also performed as these targets are closely involved with the mechanisms of action of CBD and STR, respectively, and in PTSD-induced emotional alterations. *Cnr1* relative gene expression was increased in the AMY of PTSD-like mice, and CBD, STR or its combination produced an up-regulation. It has been accepted that CBD act as an indirect agonist of CB1r by increasing anandamide (AEA) levels through the blockade of its degradation and its reuptake (Bisogno et al., 2001). In addition, in the last years some authors suggested that CBD could act also as a negative allosteric modulator of CB1r (Tham et al., 2019). On the other hand, CBD reduced the increased gene expression of *Cnr2* in the AMY of PTSD animals presenting the opposite effect in control mice, especially in combination with STR. According to the idea that CBD is an inverse agonist or an antagonist at the CB2r (Thomas et al., 2007), it is plausible that the up regulation found in control animals may be related with this mechanism. Thus, significant differences in CBD-mediated regulation of *Cnr2* between control and PTSD-like animals may depend on a differential modulation of the endocannabinoid system tone depending on the exposure or not to the animal model of PTSD.

Interestingly, CBD, STR or the combination of both drugs significantly upregulated *Slc6a4* gene expression. In control mice, only CBD or CBD plus STR treatments significantly increased *Slc6a4*. The 1A serotonin receptor (5HT1A) is one of the main targets of the actions mediated by CBD (Campos et al., 2012), mainly due to 5HT1A receptor activation (Russo et al., 2005) or allosteric modulation. Interestingly, it has been recently reported that 5HT1A receptors are involved in the induction of cortical serotonin release by CBD treatment (Linge et al., 2016). Therefore, it is possible to hypothesize that CBD reduces serotonin concentrations in the DR by upregulation of *Slc6a4* gene expression (Norris et al., 2016). Importantly, considering that CBD can interact with more than 65 different targets (Elsaid and Le Foll, 2019), additional studies are needed to further assess the underlying mechanisms involved in the effects of CBD on PTSD-related behavioral disturbances.

In conclusion, these results provide unequivocal evidence for the efficacy of CBD alone, and particularly in combination with STR, to significantly promote fear extinction and reduce anxiety-like behavior in animals exposed to an intense and long-lasting animal model of PTSD. Moreover, gene expression analyses also provide important clues regarding the short- and long-term neurobiological basis of this model of PTSD, and the mechanisms that could be underlying the pharmacological effects of CBD and STR. Some of the limitations that should be highlighted are the lack of female mice to evaluate gender-dependent effects, or the performance of a dose-response curve to have a more complete pharmacological profile of CBD and/or STR in this long-lasting animal model of PTSD. Future studies are warranted to explore the therapeutic potential of CBD for the treatment of PTSD, especially considering the increased effect when combined with STR.

## Data Availability

The raw data supporting the conclusions of this article will be made available by the authors, without undue reservation.
