# Supplementary figures and images for: Cannabidiol and Sertraline Regulate Behavioral and Brain Gene Expression Alterations in an Animal Model of PTSD
Source: Front Pharmacol. 2021 Jun 28;12:694510. doi: 10.3389/fphar.2021.694510 (PMC8273267; doi:10.3389/fphar.2021.694510)

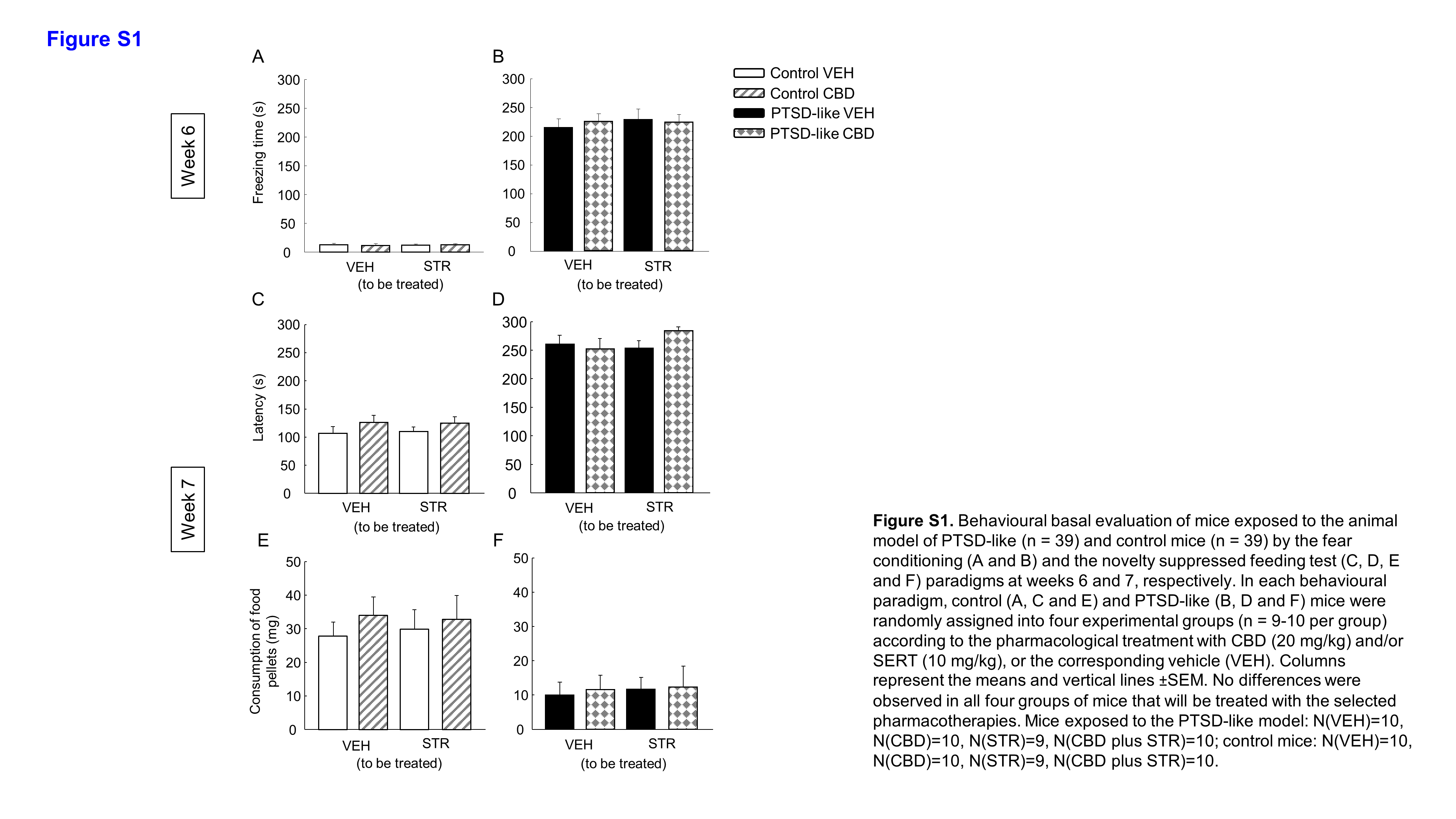

Supplement: Supplementary file 1 [file Image1.TIF]
