# Supplementary material for: Cannabidiol and Sertraline Regulate Behavioral and Brain Gene Expression Alterations in an Animal Model of PTSD
Source: Front Pharmacol. 2021 Jun 28;12:694510. doi: 10.3389/fphar.2021.694510 (PMC8273267; doi:10.3389/fphar.2021.694510)
Supplement: Supplementary file 2 [file DataSheet1.docx]

***Supplementary material***

**3. Results**

***Procedure 1: Evaluation of basal behavioural and neurobiological alterations induced by the animal model of PTSD.***

*Distribution of the treatment groups according to the basal behavioural evaluation before the beginning of the pharmacological treatment*

*Fear conditioning paradigm.* No differences were observed in the freezing time evaluated in the FC between all 4 subgroups of PTSD-exposed mice (Figure S1B, One-way ANOVA, F(3,38) = 0.17, P = 0.92), and control mice (Figure S1A, One-way ANOVA, F(3,38) = 0.05, P = 0.984) that will be treated with CBD and/or sertraline, or the corresponding vehicles.

*Novelty suppressed feeding test.* No differences were observed in the latency time and food consumption evaluated in the NSFT between all 4 subgroups of PTSD-exposed mice (Latency time, Figure S1D, One-way ANOVA, F(3,38) = 1.163, P = 0.337; Food consumption, Figure S1C, One-way ANOVA, F(3,38) = 0.08, P = 0.986) and non-PTSD mice (Latency time, Figure S1F, One-way ANOVA, F(3,38 )= 0.838, P = 0.482; Food consumption, Figure S1E, One-way ANOVA, F(3,38) = 0.241, P = 0.867) that will be treated with CBD and/or SERT, or the corresponding vehicles.

**Figure legends**

**Figure S1.** Behavioural basal evaluation of mice exposed to the animal model of PTSD-like (n = 39) and control mice (n = 39) by the fear conditioning (A and B) and the novelty suppressed feeding test (C, D, E and F) paradigms at weeks 6 and 7, respectively. In each behavioural paradigm, control (A, C and E) and PTSD-like (B, D and F) mice were randomly assigned into four experimental groups (n = 9-10 per group) according to the pharmacological treatment with CBD (20 mg/kg) and/or SERT (10 mg/kg), or the corresponding vehicle (VEH). Columns represent the means and vertical lines ±SEM. No differences were observed in all four groups of mice that will be treated with the selected pharmacotherapies. Mice exposed to the PTSD-like model: N(VEH)=10, N(CBD)=10, N(STR)=9, N(CBD plus STR)=10; control mice: N(VEH)=10, N(CBD)=10, N(STR)=9, N(CBD plus STR)=10.
